# Sarcoidosis: Key disease aspects and update on management

**DOI:** 10.1016/j.clinme.2025.100511

**Published:** 2025-09-08

**Authors:** Eamon P. McCarron, Jason Wieboldt

**Affiliations:** a Adult Inherited Metabolic Disorders, Northern General Hospital, Sheffield Teaching Hospitals NHS Foundation Trust, England, UK; b Respiratory Medicine, Daisy Hill Hospital, Southern Health and Social Care Trust (SHSCT), Newry, Northern Ireland (NI), UK

Dear Editor,

We read with interest the article by Coker and Cullen^1^ on sarcoidosis and its systemic manifestations, including the discussion of hepatosplenomegaly and salivary gland enlargement as examples of gastrointestinal involvement. As the authors rightly note, extrapulmonary disease occurs in approximately 30% of cases and can present considerable diagnostic challenges.

We would like to highlight a rare extrapulmonary manifestation not discussed in the review: appendicular sarcoidosis. Though gastrointestinal sarcoidosis is typically associated with hepatic or gastric involvement, the appendix is a recognised but extremely uncommon site of granulomatous inflammation secondary to sarcoidosis.

We report the case of a 35-year-old woman with a history of pulmonary sarcoidosis and type 1 diabetes mellitus who presented with acute right iliac fossa pain. Pulmonary sarcoidosis had been diagnosed at the age of 29 after she presented with persistent cough and night sweats, with imaging demonstrating bilateral hilar lymphadenopathy. Histological examination of transbronchial biopsy specimens at the time confirmed non-caseating granulomas and serum angiotensin-converting enzyme (ACE) levels were elevated, and she was treated with a course of systemic corticosteroids and was not receiving maintenance therapy at current presentation.

On examination, she was apyrexial but exhibited localised peritonism, and laboratory investigations revealed elevated white cell count, erythrocyte sedimentation rate (ESR) and C-reactive protein (CRP). Abdominal ultrasound (Fig. 1 A) demonstrated features consistent with acute appendicitis. She underwent appendicectomy, and histopathological examination of the resected specimen showed non-caseating granulomas with epithelioid histiocytes and Langhans-type giant cells, consistent with sarcoidosis (Fig. 1 B and C). Subsequent investigations excluded infectious aetiologies and Crohn’s disease, including negative stool cultures and polymerase chain reaction (PCR) for enteric pathogens, anti-*Saccharomyces cerevisiae* antibodies (ASCA), perinuclear anti-neutrophil cytoplasmic antibodies (pANCA) and interferon-gamma release assay (IGRA). The patient made a full recovery post-operatively and at 5 years of follow-up, the patient has developed no further gastrointestinal involvement; however, she did develop small joint arthropathy, which was treated with a short course of corticosteroids, and no other extrapulmonary manifestations.

**Fig. 1:**
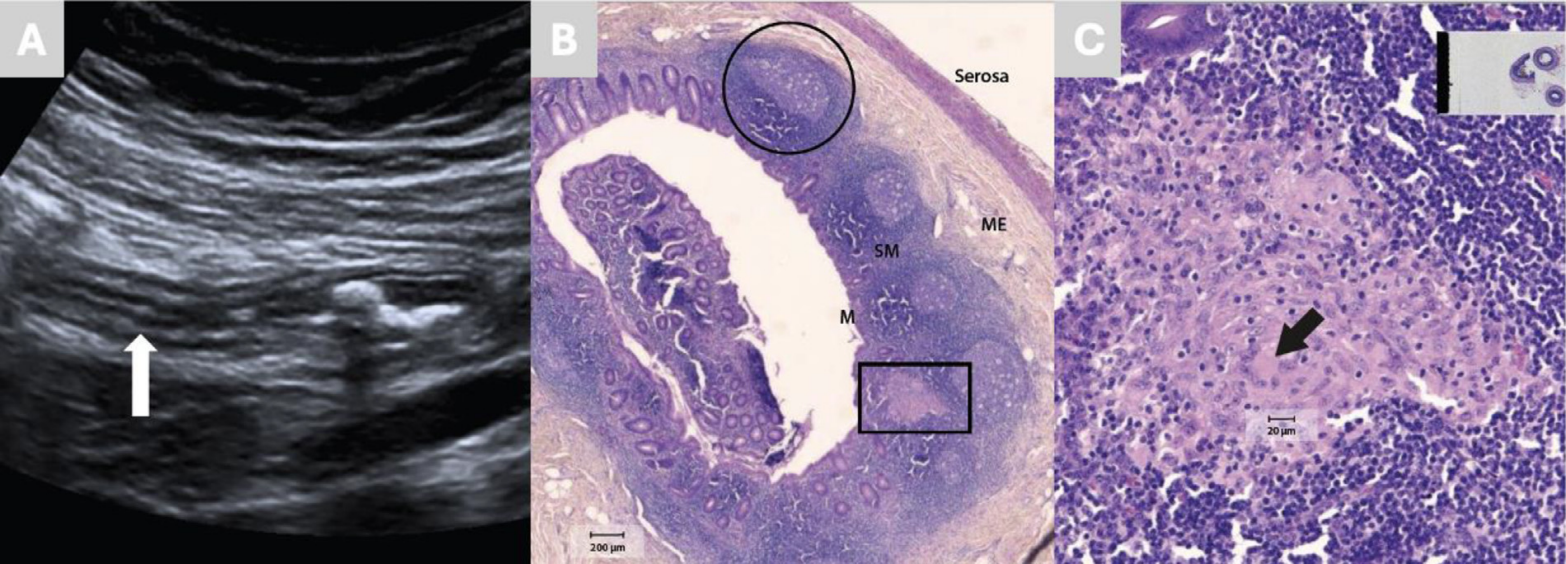
Sagittal right iliac fossa abdominopelvic ultrasound demonstrating an inflamed appendix (white arrow) (A). Low-power histopathological section of the resected appendix stained with haematoxylin and eosin (H&E) shows all layers, including the serosa and muscularis externa (ME) (B). The mucosa (M) and submucosa (SM) contain lymphoid aggregates arranged in follicles (black circle) with pale germinal centres, a normal feature of appendicular tissue. Numerous epithelioid histiocytes are present throughout the mucosa and submucosa, with one focus forming a non-caseating granuloma (black rectangle) against a background of lymphocytes and other inflammatory cells. High-power view of the non-caseating granuloma highlights Langhans-type giant cells (black arrow) (C). Magnification bars shown in histology panels.

Granulomatous appendicitis is rare, accounting for 0.1–2% of all appendicectomy histologies.² In previously published case reports, appendicular sarcoidosis has presented as acute appendicitis, often in patients with established systemic disease.^3^, ^4^, ^5^ Given the risk of perforation and the diagnostic overlap with other causes of granulomatous inflammation, such as tuberculosis, *Yersinia* and inflammatory bowel disease, histological confirmation is essential. We propose that appendicular sarcoidosis should be recognised, albeit rare, in the spectrum of extrapulmonary disease, particularly in patients with known sarcoidosis presenting with acute abdominal pain. This case supports the need for awareness among both medical and surgical teams when evaluating atypical or recurrent abdominal symptoms in this patient population.

## Consent for publication

Written consent was obtained from the patient.

## CRediT authorship contribution statement

**Eamon P. McCarron:** Writing – review & editing, Writing – original draft, Data curation, Conceptualization. **Jason Wieboldt:** Writing – review & editing, Writing – original draft, Data curation, Conceptualization.

## Declaration of competing interest

The authors declare that they have no known competing financial interests or personal relationships that could have appeared to influence the work reported in this paper.
